# Protein Machineries Involved in the Attachment of Heme to Cytochrome c: Protein Structures and Molecular Mechanisms

**DOI:** 10.1155/2013/505714

**Published:** 2013-12-23

**Authors:** Carlo Travaglini-Allocatelli

**Affiliations:** Department of Biochemical Sciences, University of Rome “Sapienza”, P.le A. Moro 5, 00185 Rome, Italy

## Abstract

Cytochromes c (Cyt c) are ubiquitous heme-containing proteins, mainly involved in electron transfer processes, whose structure and functions have been and still are intensely studied. Surprisingly, our understanding of the molecular mechanism whereby the heme group is covalently attached to the apoprotein (apoCyt) in the cell is still largely unknown. This posttranslational process, known as Cyt c biogenesis or Cyt c maturation, ensures the stereospecific formation of the thioether bonds between the heme vinyl groups and the cysteine thiols of the apoCyt heme binding motif. To accomplish this task, prokaryotic and eukaryotic cells have evolved distinctive protein machineries composed of different proteins. In this review, the structural and functional properties of the main maturation apparatuses found in gram-negative and gram-positive bacteria and in the mitochondria of eukaryotic cells will be presented, dissecting the Cyt c maturation process into three functional steps: (i) heme translocation and delivery, (ii) apoCyt thioreductive pathway, and (iii) apoCyt chaperoning and heme ligation. Moreover, current hypotheses and open questions about the molecular mechanisms of each of the three steps will be discussed, with special attention to System I, the maturation apparatus found in gram-negative bacteria.

## 1. Introduction

Cytochromes c (Cyts c) are ubiquitous heme-containing proteins involved in a variety of critical processes of cellular metabolism; since their discovery by Keilin in the early 1920s, they have been the focus of multidisciplinary scientific interests and nowadays are considered textbook proteins in biochemistry courses. However, many aspects of c-type cytochromes are still to be unveiled, from the control and fine-tuning of electron transfer reactions and heme reactivity [1–3] to the description of Cyt c folding pathways and stability [4–6]. The presence of the covalently bound heme prosthetic group dictates the functions of Cyts c, which are associated mainly with electron transfer processes in aerobic and anaerobic respiration and in photosynthesis [7, 8]; however, it is now clear that Cyts c play important roles also in other cellular processes such as H_2_O_2_ scavenging, cytochrome c oxidase assembly [9], lipid signaling [10], or apoptotic processes in the eukaryotic cells [11, 12]. This review deals with a complex and still largely unknown process, whereby the heme is covalently and stereospecifically attached to the apoprotein (apoCyt) in the cell; this posttranslational process is known as Cyt c biogenesis or Cyt c maturation. Over and above its scientific relevance, a full understanding of this posttranslational process may pave the way for future biotechnological applications, such as the design and the production *in vivo* of novel heme-proteins and biosensors endowed with innovative redox functions [13].

**Figure 1:**
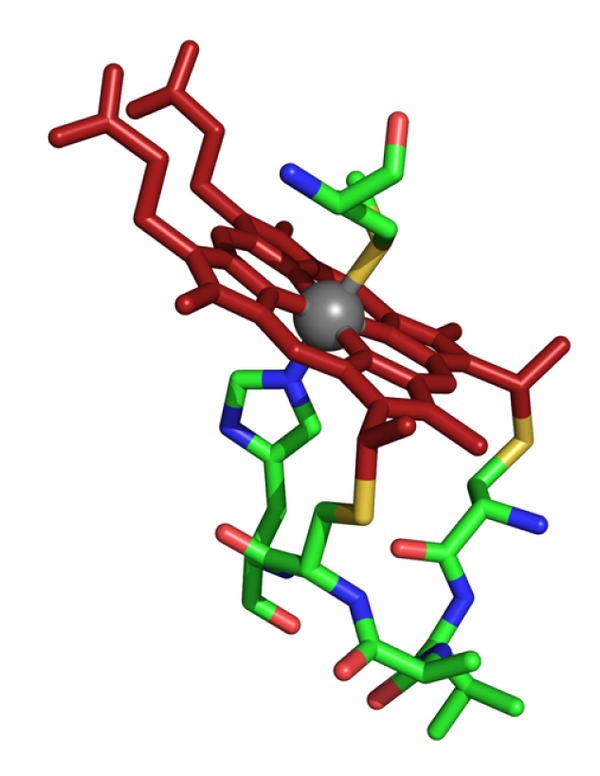
The heme-binding site typically observed in c-type cytochromes, as exemplified by a close-up view of the structure of *P. aeruginosa* Cyt c551 (Pa-Cytc; PDB 351c). The heme is shown in red, while the atoms of the residues from the heme-binding motif of Pa-Cytc (C_12_VAC_15_H) and the distal Met61 are color-coded (C: green; O: red; N: blue; S: yellow). The figure highlights the thioether bonds between the Cys12 (on the right) and the vinyl-2, and between Cys15 (on the left) and the vinyl-4. The iron atom of the heme (in gray) is axially coordinated by the distal methionine residue (Met61; shown above the heme plane) and by the proximal histidine residue (His16; shown below the heme plane).

The heme b (Fe-protoporphyrin IX) is synthesized in prokaryotes and eukaryotes along a conserved pathway with highly related enzymes and biosynthetic intermediates [14]; heme c is defined as a heme b, covalently linked to the protein by thioether bonds (Figure 1). In bacteria, heme biosynthesis occurs in the cytoplasm and the final step is the insertion of iron into protoporphyrin IX by ferrochelatase; in the eukaryotic cell, the heme biosynthetic pathway is splitted between the cytosol and the mitochondrion: here, at the level of the mitochondrial inner membrane, the ferrochelatase enzyme catalyzes the heme iron insertion. Although the heme biosynthetic pathway is well characterized, the molecular mechanism(s) underlying the process of heme trafficking across the membranes is still largely obscure (see [15, 16] for reviews on heme synthesis and trafficking in eukaryotes). In all known Cyts c, the heme is covalently linked to the apoCyt with the same stereochemistry: two thioether bonds are present between the vinyls at positions 2 and 4 of the tetrapyrrole ring of heme b and the thiols of the N- and C-terminal cysteines (Cys1 and Cys2, resp.) of a conserved heme-binding motif (C1XXC2H, where X denotes any residues). The iron atom of the Fe-protoporphyrin IX is always axially coordinated to the histidine of the heme-binding motif (on the proximal side of the heme cavity), while a methionine residue on the distal side generally represents the second axial ligand (Figure 1). C-type cytochromes may contain more than one heme c linked to the protein through different C1XXC2H motifs. From a structural point of view, Cyt c proteins define a well-defined *α*-helical fold (see SCOP—http://scop.mrc-lmb.cam.ac.uk/scop/ and CATH—http://www.cathdb.info/ protein structure databases), characterized by the presence of three *α*-helices: the N- and C-terminal *α*-helices interact each other in the native structure, while an additional *α*-helix (historically known as the 60′ helix) overlays part of the heme cavity. Since the seminal experiment of Anfinsen on horse heart Cyt c [17], it is generally accepted that Cyt c without its covalently bound heme (apoCyt) is an unfolded protein, devoid of appreciable secondary and tertiary structure and that the polypeptide chain is able to fold into its typical Cyt c structure only when the thioether bonds with the heme are formed. As it will be discussed below, these observations raise interesting questions as to how an unfolded protein such as apoCyt, is specifically recognized by the different protein components of the maturation apparatus of the cell. Recently, however, evidence has been presented that, at least in some cases, the Cyt c fold may be attained even in the absence of the heme [18], challenging our current view of the Cyt c folding mechanism [4, 19].

C-type cytochromes are synthesized in the cytoplasm (n-side of the membrane), but they exert their functions in other subcellular compartments (p-side of the membrane), that is, the periplasm of gram-negative bacteria, the bacterial extracytoplasmic space of gram-positive bacteria, the intermembrane space—IMS, of mitochondria, or the chloroplast thylakoid lumen. It is in these subcellular compartments that the heme b is covalently attached to apoCyt by the appropriate maturation apparatus. In prokaryotes, the necessary translocation of apoCyt across the membrane is carried out by the Sec machinery [26]; this apparatus, composed of the SecABDYEFG proteins, is able to translocate unfolded proteins carrying a specific targeting sequence [27]. In eukaryotes, the newly synthesized apoCyt is probably translocated into the mitochondrion via a different mechanism involving components of the TOM complex on the outer side of the membrane and the cytochrome c heme lyase, which probably acts also as an apoCyt receptor in the mitochondrial IMS [28, 29]. However, the process is not completely clear, as we still do not know whether the apoCyt is delivered to the mitochondrial matrix and then exported to the IMS [30] or it is translocated directly to the IMS via a different mechanism [31]. It should be noticed that in plants, the translocation of c-type cytochromes into the chloroplast lumen is probably independent on the heme attachment reaction [32, 33].

**Figure 2:**
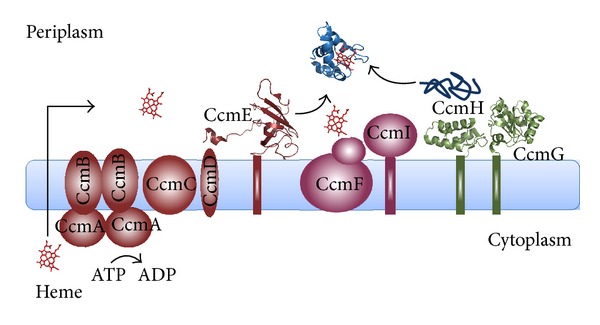
Schematic representation of the protein components of System I. Proteins involved in the heme translocation and delivery pathway are shown in light brown; proteins involved in the apoCyt thioreduction pathway are shown in green; proteins involved in apoCyt chaperoning and heme attachment processes are shown in light purple. Cyt c (the 3D structure is that of the Cyt c551 from *P. aeruginosa*), Protein Data Bank accession number 2EXV [20] and apoCyt (represented as a cartoon) are shown in blue. The translocation process of heme (shown in red) is unknown. The 3D structures of the soluble periplasmic domains of Ec-CcmE, Pa-CcmG and Pa-CcmH are shown (Protein Data Bank accession numbers are 1LIZ [21], 3KH7 [22], and 2HL7 [23], resp.). Organisms employing System I: *α*- and *γ*-proteobacteria, some *β*-proteobacteria (e.g., *Nitrosomonas*) and *δ*-proteobacteria (e.g., *Desulfovibrio*), and Deinococci and Archaea. Additionally, System I is observed in plant mitochondria and in the mitochondria of some protozoa (e.g., *Tetrahymena*).

**Figure 3:**
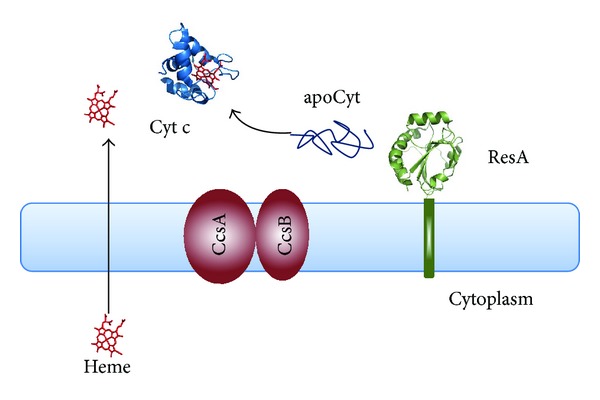
Schematic representation of the protein components of System II. Proteins involved in the heme translocation and delivery and in the apoCyt chaperoning and heme attachment processes are shown in light brown; proteins involved in the apoCyt thioreduction pathway are shown in green. Cyt c and apoCyt (represented as a cartoon) are shown in blue. The 3D structure of the soluble periplasmic domain of Bs-ResA is shown in green (Protein Data Bank accession number is 1ST9 [24]. System II is found in plant chloroplasts, in gram-positive bacteria, cyanobacteria, *ε*-proteobacteria, most *β*-proteobacteria (e.g., *Bordetella*, *Burkholderia*), and some *δ*-proteobacteria (e.g., *Geobacter*).

**Figure 4:**
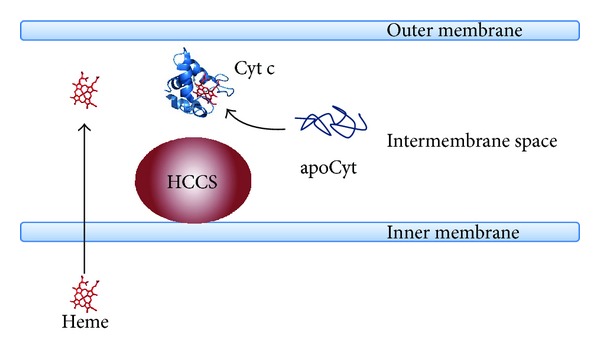
Schematic representation of System III. A single protein (HCCS) associated to the mitochondrial inner membrane is required for Cyt c maturation. The translocation process of heme (shown in red) is unknown. System III is found in the mitochondria of fungi (e.g., *S. cerevisiae*), vertebrates (e.g., human), and invertebrates (e.g.,* C. elegans*, *Drosophila*).

Despite that in all c-type cytochromes, both prokaryotic or eukaryotic, the heme is always covalently linked to the conserved CXXCH heme-binding motif, different maturation apparatuses composed of different proteins have been identified ([34, 35]; see Figures 2, 3, and 4 and Table 1). Structural and functional properties of the protein components of Systems I–III are the focus of the present review; other maturation apparatuses, involved in the unusual attachment of heme b to the protein moiety via a single thioether bond (Systems IV–VI), have been described and reviewed elsewhere [36, 37].

With the exception of system III which is present in eukaryotic cells, the distribution of the other Systems among Bacteria, Archaea, and plant cells is complicated by the observation that in many cases, the maturation machinery is not conserved [38], rendering the analysis of their evolutionary origins and relationships difficult [39–41]. In *α*- and *γ*-proteobacteria, in some *β*- and *δ*-proteobacteria, in Archaea and in the mitochondria of plants and algae, Cyt c maturation is carried out by a set of eight or nine proteins belonging to System I [42] (Figure 2); in gram-positive bacteria, in cyanobacteria, in the chloroplasts of plants and algae, in *ε*-, *β*- and some *δ*-proteobacteria the Cyt c maturation process is carried out by three or four proteins belonging to System II [43, 44] (Figure 3), while System III occurs in mitochondria of fungi, metazoans, and some protozoa [16, 45] (Figure 4). The observation that in plants, three systems are present (System I in mitochondria, System III in the p-side of the thylakoid membrane, and System IV in the n-side of the thylakoid membrane [16, 38]) makes the classification and the distribution of the different maturation systems even more difficult. With the exception of System III, which is apparently composed of a single protein able to carry out the different tasks of the Cytc maturation process (see below), the various proteins of Systems I and II carry out different functions, including the translocation and delivery of heme b from the cytoplasm where it is synthesized to the relevant subcellular compartment, the chaperoning of apoCyt and the reduction of its disulfide, the formation of the covalent bonds between the heme b and the CXXCH heme-binding motif of the apoprotein (Table 1). The complexity of System I, compared to the protein composition of other Cyt c maturation systems, has long been discussed; in particular, it has been proposed that a possible explanation is to be found in the ability evolved by organisms employing System I to utilize lower levels of endogenous heme than those necessary for organisms which evolved Systems II or III [46].

## 2. System I

The proteins belonging to System I (named CcmABCDEFGH(I), from Cytochrome c maturation), are membrane proteins exposing their soluble domains (when present) into the periplasm (Figure 2). All of these proteins are encoded by a single operon in *α*-, *β*-, and *γ*-proteobacteria [42, 47]. The availability of the entire Ccm operon in a single plasmid (pEC86; [48]) greatly facilitated the heterologous over-expression of many c-type cytochromes in *E. coli* [49]. Apparently, c-type cytochromes specificity of the Ccm apparatus is rather low; indeed, in an attempt to characterize the minimal sequence requirements of the apoCyt polypeptide recognized by System I, it was shown that this complex multiprotein apparatus is able to attach the heme even to short, microperoxidase-like peptides carrying the CXXCH motif [50].

As discussed below, different studies, mainly carried out by immunoprecipitation experiments, suggest that the Ccm proteins may be assembled in the bacterial membrane in a maturase multiprotein complex(es). However, the existence and/or stoichiometry of these complexes remains to be determined, either because of the experimental difficulties in handling membrane protein complexes, or because it is possible that these complexes are unstable and only transiently populated. Independently from their functional existence in the bacterial periplasm as independent units or as components of a multisubunit complex, it is clear that each of the Ccm proteins plays a different role, from the transport and chaperoning of the heme cofactor, to the necessary reduction of the disulfide bond between the sulfur atoms of the two Cys residues of the conserved CXXCH motif of apoCyt, and finally to the catalysis of covalent heme attachment. An additional interesting aspect is that, over and above their role in the biogenesis of Cytc, there is also evidence that inactivation of some ccm genes induces phenotypes that cannot be explained only in terms of absence of synthesis of Cyt c; all of these pleiotropic effects are linked to impairment of heme and/or iron trafficking in the periplasm [47]. In particular, it has been recently shown that, in *α*- and *γ*-proteobacteria (including the human opportunistic pathogen *P. aeruginosa*), mutations in the ccmC, ccmI and ccmF genes induce phenotypes such as reduced pyoverdine production, reduced bacterial motility or impaired growth in low-iron conditions ([51] and refs. therein). These observations, suggesting that Ccm proteins perform additional functions critical for bacterial physiology, growth and virulence, provide a rationale to explain why bacteria, at variance with the eukaryotic cell, have evolved a metabolically expensive operon to accomplish an apparently simple task such as heme ligation to apoCyt. Novel hypotheses addressing these aspects and awaiting experimental investigation include (i) the utilization of Ccm-associated heme for additional cellular processes besides attachment to apoCyt, (ii) trafficking a nonheme compound through the Ccm system required for iron acquisition such a siderophore, (iii) the Ccm inactivation-dependent accumulation of heme b, a photoreactive molecule whose degradation leads to reactive oxygen species, and (iv) the destructive effect on [Fe-S] clusters of ferrisiderophores reductases.

In the following, the structural (when known) and functional properties of the different components of the System I maturation apparatus will be discussed, dissecting the Cytc maturation process into three main functional steps: heme translocation and delivery, apoCyt thioreductive pathway, and apoCyt chaperoning and heme ligation (a similar modular description can also be found elsewhere (see [34, 52])). However, it should be remembered that in many cases the proteins involved in the three steps are not uniquely assigned to a specific module, as they interact with each other; moreover, it has been shown that, in some cases, more than one system can be present [41]. This modular organization of Cyt c biogenesis should therefore be intended only as a way to simplify the description of an overall, highly integrated process. For each of the three functional steps, presentation of the structural and functional properties of the different protein components is followed by a discussion of the proposed molecular mechanism(s).

### 2.1. System I: Components of the Heme Translocation and Delivery Pathway

CcmA and CcmB proteins show the typical sequence features of the ABC (ATP binding Cassette) transporter family and therefore these components of System I were initially considered as the proteins responsible for the translocation of the newly synthesized heme from the cytoplasm to the periplasmic space. ABC transporters are ubiquitous, multidomain integral membrane proteins that translocate a large variety of substrates across cellular membranes using ATP hydrolysis as a source of energy; they are generally composed of a transmembrane (TM) domain and a conserved cytosolic nucleotide-binding domain [53].

CcmA is a cytoplasmic soluble protein, representing the nucleotide-binding domain of the hypothetical ABC transporter; according to this hypothesis, its sequence contains a nucleotide-binding domain and Walker A and B motifs for ATP hydrolysis [46]. It has also been shown that CcmA possesses ATPase activity *in vitro* and that the protein is associated with the membrane fraction only when CcmB is also present [54].

CcmB and CcmC are both integral membrane proteins predicted to contain six TM helices. CcmC contains a short WWD domain in its second periplasmic domain and belongs to the heme handling protein family (HHP) [55]. WWD domains are short, tryptophan-rich, aminoacid stretches with the conserved WGX*ϕ*WXWDXRLT sequence (where *ϕ* represents an aromatic amino acid residue and X represents any residue) [40, 56]; it has been proposed that proteins containing WWD domains are involved in heme-binding and, as we will see below, a WWD domain is also present in the CcmF protein, another crucial heme-binding protein of System I. CcmC also contains two absolutely conserved histidine residues in its first (between TM helices 1 and 2) and third (between TM helices 5 and 6) periplasmic domains. An attractive hypothesis, still requiring experimental proof, is that the hydrophobic residues within the tryptophan-rich motif provide a platform for the binding of heme, whereas the two conserved His residues (H60 and H184 in *E. coli* CcmC) act as axial heme ligands [57]; this hypothesis is strengthened by the observation that CcmC indeed interacts directly with heme [58, 59]. Immunoprecipitation experiments have shown that in *E. coli*, CcmABC proteins form a multiprotein complex with a CcmA_2_CcmB_1_CcmC_1_ stoichiometry, confirming that these components form an ABC-type transporter complex with unusual functional properties associated to the release of holoCcmE from CcmC [41, 46], rather than to heme transport *per se*. CcmC is an interesting protein, worth of future experimental efforts, as it is known that in some pathogenic bacteria, CcmC mutations are associated to specific phenotypes apparently not related to Cyt c maturation, such as siderophore production in *Paracoccus* and *Pseudomonas* [51] and iron utilization in *Legionella* [60].

Limited information is available about the structure and function of CcmD, which appears to be a small membrane protein (about 70 aminoacid residues) with no conserved sequence features, whose topology is currently debated; contrary to the original proposal [61], additional experiments have shown that in *E. coli* and *R. capsulatus,* CcmD is an integral membrane protein composed of a single TM helix, a periplasmic-oriented N-terminus, and a cytoplasmic-oriented C-terminus [62]. Immunoprecipitation experiments indicate that CcmD interacts with the CcmA_2_CcmB_1_CcmC_1_ complex; even if it is not essential for heme transfer and attachment from CcmC to CcmE, CcmD is strictly required for the release of holoCcmE from the ABC transporter [61, 62].

CcmE is a heme-binding protein, discovered as an essential System I component as early as the late 1990's [63]. CcmE is a monotopic membrane protein, anchored to the membrane via its N-terminal TM segment and exposing its active site to the periplasm; it is the only Ccm component of the heme trafficking and delivery module of System I for which a three-dimensional structure is available ([21] PDB: 1SR3; [64] PDB: 1LM0). The 3D structure of the apo-state (without bound heme) consists of a six-stranded antiparallel *β*-sheet, reminiscent of the classical OB-fold [65] with N- and C-terminal extensions. CcmE can be considered a “heme chaperone,” as it protects the cell from a potentially dangerous compound by sequestering free heme in the periplasm [66]; it is thought to act as an intermediate in the heme delivery pathway of Cytc maturation. The structure of apoCcmE showed no recognizable heme-binding cavities and, in the absence of a 3D structure of CcmE with bound heme (holoCcmE), the heme-binding region could only be predicted by *in silico* modeling. It is generally believed that the heme in holoCcmE is solvent exposed, but recent mutagenesis experiments challenged this view [67]. The unusual covalent bond between the nitrogen atom of a histidine residue present in the conserved VLAKHDE motif located in a solvent exposed environment (H130 in *E. coli* CcmE) and a *β*-carbon of one of the heme vinyl groups has been described in great detail by NMR spectroscopy [68]. Recently, it was shown that CcmE proteins from the proteobacteria *D. desulfuricans* and *D. vulgaris* contain the unusual CXXXY heme-binding motif, where the Cys residue replaces the canonical His binding residue. NMR solution structure of *D. vulgaris* CcmE (PDB: 2KCT) revealed that the proteins adopt the same OB-fold characteristic of the CcmE superfamily. Contrary to what reported for the *D. desulfuricans* CcmE [69], the homologous protein from *D. vulgaris* binds ferric heme noncovalently through the conserved C127 residue [70]. An additional conserved residue in CcmE proteins is Tyr134, which was shown to provide a coordination bond to the heme iron of holoCcmE [71, 72] once it is released from CcmABCD complex [36], as discussed below.

### 2.2. System I: Heme Translocation and Delivery Pathway Mechanisms

We still do not know how the b heme is translocated from the cytoplasm (where it is synthesized) to the periplasm, where Cyt c maturation occurs. Different mechanisms such as translocation through a protein channel or free diffusion across the membrane have been proposed [73]. The CcmAB proteins show structural features typical of the ABC transporters and for these reasons, they were originally hypothesized to be involved in the heme b translocation process [57, 63, 74, 75]. However, it is now clear that an alternative process must exist, since it has been shown that periplasmic b-type cytochromes can be produced in the absence of Ccm proteins [76] and that inactivation of the ATPase activity of CcmA does not abolish heme accumulation in the periplasm [46, 54]. We have now evidence that CcmC has the ability to bind heme at its WWD domain present in the second periplasmic domain, but it is still not clear if this membrane protein acts as a protein channel for heme translocation, or simply collects it in the periplasm [77].

Another important aspect concerns the oxidation state of the heme iron during translocation and delivery processes; indeed, this property of the heme iron may determine the reaction mechanism by which the unusual CcmE H130 nitrogen is covalently linked to the vinyl *β*-carbon of the heme (see [36] for a detailed discussion of this topic). Based on mutagenesis studies on CcmC [59], a model has been presented whereby oxidized heme is bound to CcmC only in the presence of CcmE, forming a ternary complex. Both CcmC and CcmE provide critical residues for heme-binding: the two conserved His residues (H60 and H180, coordinating the heme iron) and the WWD domain of CcmC and His130 of CcmE, forming the unusual covalent bond with heme vinyl-2 [59]. The ATPase activity of CcmA is then required to release holoCcmE from the CcmABCD complex, a process that depends also on the presence of CcmD [46, 54]. It should be noticed that purified holoCcmE alone or in the CcmCDE complex [36, 59] contains the heme iron in the oxidized state, an observation that is apparently in contrast with the fact that the heme must be in its reduced state before attachment to apoCyt can occur. Although the oxidation state of the heme iron is currently debated [36, 78], it is possible that CcmF, which was recently shown to contain a heme b cofactor, may act as specific heme oxidoreductase (see Section 2.5).

### 2.3. System I: ApoCyt Thioreduction Pathway Components

The periplasm can be considered a relatively oxidizing environment, due to the presence of an efficient oxidative system composed of the DsbAB proteins [79, 80]. DsbA is a highly oxidizing protein (*E*_0_′ = 120 mV) that is responsible for the introduction of disulfide bonds into extracytoplasmic proteins [81]. On the basis of the results obtained on *E. coli* dsbA deletion mutants that are unable to synthesize c-type cytochromes [82, 83], it was generally accepted that formation of the intramolecular disulfide bond in apoCyt was a necessary step in the Cyt c biogenesis. However, only reduced apoCyt is clearly competent for heme ligation. It is possible that this seemingly paradoxical thioreduction process has evolved in order to protect the apoCyt from proteolytic degradation, aggregation, and/or formation of intermolecular disulfide bonds with thiols from other molecules (see also Section 3.1 for a discussion about this aspect in System II). Recently, however, an analysis of c-type cytochromes production in several *E. coli* dsb genes deletion strains led to the hypothesis that DsbA is not necessary for Cyt c maturation and that heme ligation to apoCyt and apoCyt oxidation pathways is alternative, competing processes [84].

In gram-negative bacteria, a thioreduction pathway has evolved to specifically reduce the oxidized apoCyt substrate, which includes the Ccm proteins CcmG and CcmH. The necessary reducing power is transferred from the cytoplasmic thioredoxin (TRX) to CcmG via DsbD, a large membrane protein organized in three structural domains: an N-terminal periplasmic domain with a IgG-like fold (nDsbD), a C-terminal periplasmic domain with a thioredoxin-like (TRX-like) fold (cDsbD), and a central domain composed of eight TM helices [85]. Each of these domains contains a pair of Cys residues and transfer electrons via a cascade of disulfide exchange reactions, making DsbD a “redox-hub” in the periplasm, performing disulfide bond exchange reactions with different oxidized proteins [79]. In particular, a combination of X-ray crystallography experiments and kinetic analyses showed that electrons are transferred from the cytoplasmic TRX to the membrane domain of DsbD, followed by reduction of cDsbD and finally of nDsbD which is the direct electron donor to CcmG [85].

CcmG is a membrane-anchored protein, linked to the membrane via an N-terminal TM helix and exposing its soluble TRX-like domain in the periplasm. The 3D structure of the TRX-like domain of CcmG from different bacteria has been solved by X-ray crystallography (*E. coli*: PDB 1Z5Y [85]; PDB 2B1 K [86]; *B. japonicum*: PDB 1KNG [87]; *P. aeruginosa*: PDB 3KH7, 3KH9 [22]) and is generally well conserved, as proved by the low RMSD (0.8 Å between *Pa-*CcmG and Ec-CcmG; 1.35 Å between Pa-CcmG and Bj-CcmG). Although all these proteins adopt a TRX-like fold and contain the redox-active motif CXXC in the first *α*-helix, they are inactive in the classic insulin reduction assay [75, 88]; CcmG proteins are therefore considered specific thiol-oxidoreductase, able to recognize and selectively interact only with their upstream and downstream binding partners in the thioreduction process leading to reduced apoCytc. Looking at the 3D structure of the periplasmic domain of the prototypical Pa-CcmG, it is possible to identify the *βαβ* and *ββα* structural motifs of the TRX fold linked by a short *α*-helix and forming a four-stranded *β*-sheet surrounded by three helices; the protein contains an additional N-terminal extension (residues 26–62) and a central insert (residues 102–123). The redox-active motif of Pa-CcmG (CPSC) is located in the first *α*-helix of the TRX fold, as usually observed in all TRX-like proteins. As for any molecular machinery, where each component must recognize and interact with more than one target (i.e., the substrate and the other components of the apparatus), an open question concerns the mechanism whereby CcmG is able to recognize its different partners. The availability of the crystal structures of Pa-CcmG both in the oxidized (2.2 Å resolution) and reduced state (1.8 Å resolution) [22] allowed highlighting the structural similarity between the two redox states (Rmsd of the C*α* atoms in the two redox forms is 0.19 Å) and therefore to exclude structural rearrangement as the mechanism used by Pa-CcmG to discriminate between reduced (such as the nDsbD domain) and oxidized partners (Pa-CcmH and/or apoCyt).

The standard redox potential of Pa-CcmG (*E*_0_′ = 0.213 V at pH 7.0; [22], as well as that of Ec-CcmG (*E*_0_′ = 0.212 V [86]), indicates that these proteins act as mild reductants in the thioreductive pathway of Cytc biogenesis. However, the function of thiol-oxidoreductases obviously depends on the pK_*a*_ values of their activesite Cys residues. The pK_*a*_ of CysX (6.13 ± 0.05) and CysY (10.5 ± 0.07) are consistent with the pK_*a*_ values measured in different TRXs, where the active N-terminal Cys residue has a pK_*a*_ close to pH 7.0, whereas the C-terminal Cys has a much higher pK_*a*_ [89, 90]. Such a large difference between the two pK_*a*_ values in the TRX family is functionally relevant, because it allows the N-terminal Cys to perform the nucleophilic attack on the target disulfide, while the C-terminal Cys is involved in the resolution of the resulting mixed-disulfide [90].

**Figure 5:**
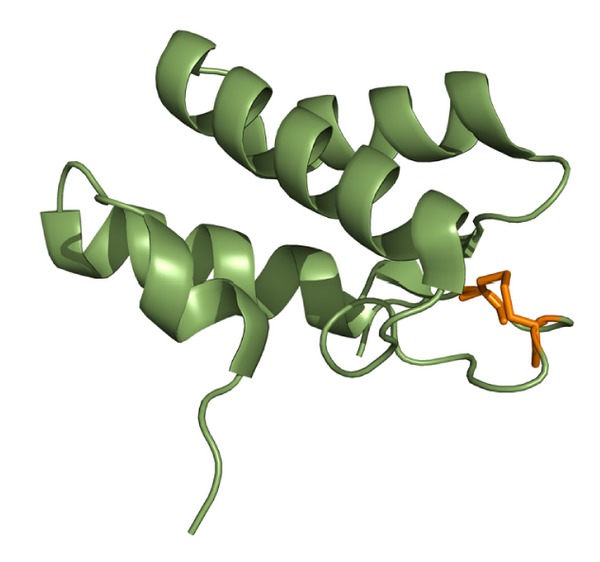
Three-dimensional structure of Pa-CcmH shown in ribbon representation. The figure shows the three-helix bundle forming the characteristic fold of Pa-CcmH. The active site disulfide bond between residues Cys25 and Cys28 in the long loop connecting helices *α*-helix1 and *α*-helix 2 is highlighted in yellow.

CcmH is the other component of System I involved in the reduction of apoCyt. Notably, CcmH proteins from different bacterial subgroups may display structural variability; indeed, while in *E. coli* Ec-CcmH is a bipartite protein characterized by two soluble domains exposed to the periplasm and two TM segments, CcmH from *P. aeruginosa* (Pa-CcmH) is a one-domain redox-active protein, anchored to the membrane via a single TM helix and homologous to the N-terminal redox-active domain of Ec-CcmH. Surprisingly, the 3D structure of the soluble periplasmic domain of Pa-CcmH revealed that it adopts a peculiar three-helix bundle fold strikingly different from that of canonical thiol-oxidoreductases (Figure 5; PDB: 2HL7; [23]). The N-terminal domain of Ec-CcmH was also shown to have the same 3D structure, although helix-swapping and dimerization have been observed in this case (PDB: 2KW0; [91, 92]). The conserved redox-active motif (LRCPKC) is located in the loop connecting helices 1 and 2; close to the activesite, the crystal structure reveals the presence of a small pocket on the surface of Pa-CcmH surrounded by conserved hydrophobic and polar residues, which could represent the recognition site for the heme-binding motif of apoCyt.

Concerning the functional properties of this unusual thiol-oxidoreductase, it is interesting to note that its standard redox potential (*E*_0_′ = 0.215 V) [23] is similar to that obtained for Pa-CcmG. This observation stands against the linear redox cascade hypothesis, whereby CcmG reduces CcmH. While in the canonical redox-active CXXC motif of the TRX family, the N-terminal Cys is always solvent exposed, in CcmH proteins, the arrangement of the two Cys residues is reversed: the N-terminal Cys residue is buried, whereas the C-terminal Cys residue is solvent exposed. On the basis of this observation, it was suggested that, different from the canonical TRX redox mechanism, CcmH proteins perform the nucleophilic attack on the apoCyt disulfide via their C-terminal Cys residue [23]. This mechanism, which is in agreement with the mechanism proposed earlier for Ec-CcmH on the basis of mutational-complementation studies [93, 94], is substantiated by the peculiar pK_*a*_ values of the active site Cys residues of Pa-CcmH which were found to be similar for both cysteines (8.4 ± 0.1 and 8.6 ± 0.1; [23]). Again, this is different from what is generally observed in the case of TRX proteins, where the pK_*a*_ value of the Cys residue performing the initial nucleophilic attack is significantly lower than the pK_*a*_ value of the Cys residue responsible for the resolution of the intermediate mixed-disulfide. It is tempting to speculate that the unusual pK_*a*_ values of the Pa-CcmH active site thiols may ensure the necessary specificity of this component of the Ccm apparatus toward the CXXCH motif of the apoCyt substrate.

### 2.4. System I: ApoCyt Thioreduction Pathway Mechanism

**Figure 6:**
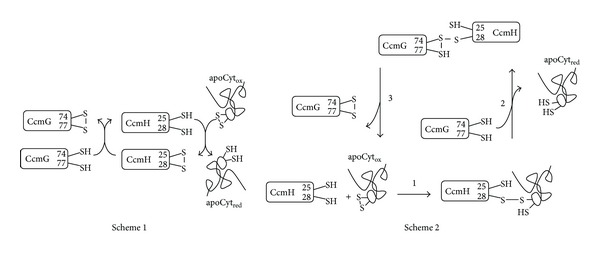
**: **Alternative thioreduction pathways which may be operative in System I and hypothesized on the basis of structural and functional characterization of the redox-active Ccm proteins from *P. aeruginosa* [22, 23, 25]. Scheme 1 is a linear redox cascade whereby CcmG is the direct reductant of CcmH, which reduces oxidized apoCyt. Scheme 2 envisages a more complex scenario involving the formation of a mixed-disulfide complex between CcmH and apoCyt (Step 1). This complex is the substrate for the attack by reduced CcmG (Step 2) that liberates reduced apoCyt. The resulting disulfide bond between CcmH and CcmG is then resolved by the free Cys thiol of CcmG (probably Cys77 in Pa-CcmG). Adapted from [25].

Although we know that CcmG and CcmH are the redox-active components of System I involved in the thioreductive pathway of Cyt c biogenesis, not only an accepted mechanism for the reduction of apoCyt disulfide bond is still lacking, but also the absolute requirement of such a process is now debated [38, 84]. Focusing our attention on the reduction of the apoCyt internal disulfide, at least two mechanisms can been hypothesized, which involve either a linear redox cascade of disulfide exchange reactions or a nonlinear redox process involving transient formation of a mixed-disulfide complex, as depicted in Figure 6 and Schemes 1 and 2, respectively.

Both the thiol-disulfide exchange mechanisms depicted in Figure 6 suggest that CcmH is the direct reductant of the apoCytc disulfide; however, even if immunoprecipitation experiments failed to detect the formation of a mixed-disulfide complex between apoCyt and CcmH proteins [95], some *in vitro* evidence supporting the formation of such a complex has been presented. In particular, it has been shown that *Rhodobacter capsulatus* and *Arabidopsis thaliana* CcmH homologues (Rc-CcmH and At-CcmH) are able to reduce the CXXCH motif of an apoCyt-mimicking peptide [75, 96]. In the latter case, yeast two-hybrid experiments carried out on At-CcmH, indeed revealed an interaction between the protein and a peptide mimicking the *A. thaliana* Cyt c sequence. In the case of *Pa*-CcmH, FRET kinetic experiments employing a Trp-containing fluorescent variant of the protein and a dansylated nonapeptide encompassing the heme-binding motif of *P. aeruginosa* cytochrome c551 (dans-KGCVACHAI) [23] allowed to directly observe the formation of the mixed-disulfide complex and to measure the off-rate constant of the bound peptide. The results of these *in vitro* binding experiments allowed to calculate an equilibrium dissociation constant which combines an adequate affinity (low *μ*M) with the need to release efficiently reduced apoCyt to other component(s) of the System I maturase complex [23]. More recently, the results obtained by FRET binding experiments carried out with single Cys-containing mutants of Pa-CcmH and Pa-CcmG [25] substantiated the hypothesis depicted in Scheme 2 (Figure 6). Altogether, these structural and functional results suggest that the thioreduction pathway mechanism leading to reduced apoCyt is better described by Scheme 2 and that reducing equivalents might not be transferred directly from CcmG to apoCyt as depicted in Scheme 1. According to Scheme 2, reduced CcmH (a non-TRX-like thiol-oxidoreductase) specifically recognizes and reduces oxidized apoCyt via the formation of a mixed-disulfide complex, which is subsequently resolved by CcmG. The resulting disulfide bond between CcmH and CcmG is then resolved by the free Cys thiol of CcmG (probably Cys77 in Pa-CcmG).

However, further *in vitro* experiments with CcmH and apoCyt single Cys-containing mutants are needed to unveil the details of the thioreduction of oxidized apoCyt by CcmH. In particular, it would be crucial to identify the Cys residue of apoCyt that remains free in the apoCyt-CcmH mixed-disulfide complex intermediate (see Scheme 2 and Section 2.6 below) and available to thioether bond formation with one of the heme vinyl groups. Clearly, structure determination of the trapped mixed-disulfide complexes between CcmH, CcmG, and apoCyt (or apoCyt peptides) would provide key information for our understanding of this specialized thioreduction pathway mechanism.

### 2.5. System I: ApoCyt Chaperoning and Heme Attachment Components

The reduced heme-binding motif of apoCyt is now available to the heme ligation reaction. However, the molecular mechanism whereby the Ccm machinery catalyzes or promotes the formation of the heme-apoCyt covalent bonds is still largely obscure, representing the most important goal in the field. Past observations and recent experiments suggest that CcmF and CcmI, possibly together with CcmH, are involved in these final steps [16, 34, 36].

CcmF is a large integral membrane protein of more than 600 residues, belonging to the heme handling protein family (HHP; [55]) and predicted to contain 10–15 TM helices (note that some discrepancy exists as to the number of TM helices predicted by computer programs and those predicted on the basis of phoA and lacZ fusion experiments; [40, 97]), a conserved WWD domain, and a larger domain devoid of any recognizable sequence features, both exposed to the periplasm. Only recently, *E. coli* CcmF (Ec-CcmF) has been overexpressed, solubilized from the membrane fraction, and spectroscopically characterized *in vitro* [36, 41]. Surprisingly, the biochemical characterization of recombinant Ec-CcmF allowed to show that the purified protein contains heme b as cofactor in a 1 : 1 stoichiometry; this observation led to the hypothesis that, in addition to its heme lyase function, Ec-CcmF may act as a heme oxidoreductase. In particular, it is possible that the heme b of Ec-CcmF may act as a reductant for the oxidized iron of the heme bound to CcmE [41]; indeed, the *in vitro* reduction of Ec-CcmF by quinones has been experimentally observed, strengthening the hypothesis about the quinol:heme oxidoreductase function of this elusive protein. The structural model proposed for Ec-CcmF predicts 13 TM helices and, notably, the location of the four completely conserved His residues: according to the model, two of them (His173 and His303) are located in periplasmic exposed loops next to the conserved WWD domain, which is believed to provide a platform for the heme bound to holoCcmE, while His261 is located in one of the TM helices and it is predicted to act as an axial ligand to the heme b of Ec-CcmF; the other conserved His residue (H491) could provide the second axial coordination bond to the heme, although this has not been experimentally addressed. This model of Ec-CcmF therefore envisages that this large membrane protein is characterized by two heme-binding sites: one of them is embedded in the membrane and coordinates a heme b prosthetic group necessary to reduce the CcmE-bound heme hosted in the second heme-binding site and constituted by its WWD domain.

It is interesting to note that in plants mitochondria the CcmF ortholog appears to be split into three different proteins (At-CcmFN1, At-CcmFN2 and At-CcmFC), possibly interacting each other [16]. Since each of these proteins is similar to the corresponding domain in the bacterial CcmF ortholog, this observation may provide useful information in the design of engineered fragments of bacterial CcmF proteins, amenable to structural analyses.

The other System I component, which is generally believed to be involved in the final steps of Cyt c maturation, is CcmI. As stated above, the ccmI gene is present only in some Ccm operons, while in others, the corresponding ORF is present within the ccmH gene (as in *E. coli*). The functional role of CcmI in Cytc biogenesis is revealed by genetic studies, showing that in *R. capsulatus* and *B. japonicum,* inactivation of the ccmI gene leads to inability to synthesize functional c-type cytochromes [98, 99]. In *R. capsulatus* and *P. aeruginosa*, the CcmI protein (Rc-CcmI and Pa-CcmI, resp.) can be described as being composed of two domains, starting from the N-terminus: a first domain composed of two TM helices connected by a short cytoplasmic region and a large periplasmic domain. Structural variations may be observed among CcmI members from different bacteria; indeed, multiple sequence alignment indicates that the cytoplasmic region of Rc-CcmI contains a leucine zipper motif, which is not present in the putative cytoplasmic region of Pa-CcmI [100–102]. Surprisingly, no crystallographic structure is available up to now for the soluble domain of any CcmI protein, with the exception of the ortholog protein NrfG from *E. coli*, (Ec-NrfG) [103]. This protein is necessary to attach the heme to the unusual heme-binding motif CWSCK (where a Lys residue substitutes the conserved His) present in NrfA, a pentaheme c-type cytochrome [103, 104]. According to secondary structure prediction methods [105], it has been proposed that the periplasmic domain of Pa-CcmI is composed of a N-terminal *α*-helical region containing at least three TPR motifs connected by a disordered linker to a *α*-*β* C-terminal region. Multiple sequence analyses and secondary structure prediction methods show that the TPR region of Pa-CcmI can be successfully aligned with many TPR-containing proteins, including Ec-NrfG [106].

### 2.6. System I: ApoCyt Chaperoning and Heme Attachment Mechanisms

TPR domain-containing proteins are common to eukaryotes, prokaryotes, and *archaea*; these proteins are generally involved in the assembly of multiprotein complexes and to the chaperoning of unfolded proteins [103, 107]. It is therefore plausible that CcmI (or the TPR C-terminal domain of Ec-CcmH) may act to provide a platform for the unfolded apoCyt, chaperoning it to the heme attachment site, presumably located on the WWD domain of CcmF. CcmI may thus be considered a component of a membrane-integral multisubunit heme ligation complex, together with CcmF and CcmH, as experimentally observed by affinity purification experiments carried out with Rc-CcmFHI proteins [97, 99, 108]. According to the proposed function of CcmI, a critical requirement is represented by its ability to recognize different protein targets over and above apoCyt, such as CcmF and CcmH. However, until now, direct evidence has been presented only for the interaction of CcmI with apoCyt, but the possibility remains that CcmFHI proteins interact each other via their TM helices and not via their periplasmic domains. Interestingly, both for Pa-CcmI [106] or Rc-CcmI [99], CD spectroscopy experiments carried out on the CcmI:apoCyt complex highlighted major conformational changes at the secondary structure level. It is tempting to speculate, on the basis of these results, that *in vivo* the folding of apoCyt may be induced by the interaction with CcmI. In the case of *P. aeruginosa* System I proteins, the binding process between Pa-CcmI and its target protein, apoCyt c551 (Pa-apoCyt), has been studied both at equilibrium and kinetically [106]; the *K*_*D*_ measured for this interaction (in the *μ*M range) appeared to be low enough to ensure apoCyt delivery to the other components of the Ccm machinery. Clearly, a major question concerns the molecular determinants of such recognition process; interestingly, both affinity coprecipitation assays [99] and equilibrium and kinetic binding experiments [106] highlighted the role played by the C-terminal *α*-helix of Cyt c. Similar observations have been made for the interaction of Ec-NrfG with a peptide mimicking NrfA, its apoCyt substrate [103]; in this case, isothermal titration calorimetry (ITC) experiments indicate that the TPR-domain of NrfG serves as a binding site for the C-terminal motif of NrfA. Altogether, these observations are in agreement with the fact that TPR proteins generally bind to their targets by recognizing their C-terminal region [107].

The CcmI chaperoning activity has been experimentally supported for the first time in the case of Pa-CcmI by citrate synthase tests [106]: it has been proposed that the observed ability to suppress protein aggregation *in vitro* may reflect the capacity of CcmI to avoid apoCyt aggregation *in vivo*. Still another piece of the Cyt c biogenesis puzzle has been added recently by showing that Rc-CcmI is able to interact with apoCcmE, either alone or together with its substrate apoCyt c2, forming a stable ternary complex in the absence of heme [109]. This unexpected observation, obtained by reciprocal copurification experiments, provides supporting evidence for the existence of a large multisubunit complex composed of CcmFHI and CcmE, possibly interacting with the CcmABCD complex. It is interesting to note that, while in the case of the CcmI:apoCyt recognition different studies highlighted the crucial role of the C-terminal helical region of apoCyt (see above), in the case of the apoCcmE: apoCyt recognition, the N-terminal region of apoCyt seems to represent a critical region.

It is generally accepted that CcmF is the Ccm component responsible for heme covalent attachment to apoCyt; however, as discussed above, it is possible that this large membrane protein plays such a role only together with other Ccm proteins such as CcmH and CcmI. Moreover, as recently discovered by Kranz and coworkers [36, 41], CcmF may also act as a quinole:heme oxidoreductase, ensuring the necessary reduction of the oxidized heme b bound to CcmE. Why it is necessary that the heme iron be in its reduced state rather than in its oxidized state is not completely clear, although it is possible that this is a prerequisite to the mechanism of thioether bond formation [110]. According to current hypotheses, it is likely that the periplasmic WWD domain of CcmF provides a platform for heme b binding. Sanders et al. and Verissimo et al. [34, 109] have presented a mechanistic view of the heme attachment process, which takes into account all the available experimental observations on the different Ccm proteins. According to this model, stereospecific heme ligation to reduced apoCyt occurs because only the vinyl-4 group is available to form the first thioether bond with a free cysteine at the apoCyt heme-binding motif, since the vinyl-2 group is involved (at least in the Ec-CcmE) in the covalent bond with His130 of CcmE [67]. However, experimental proof for this hypothesis requires a detailed investigation of the apoCyt thioreduction process catalyzed by CcmH (see Section 2.4).

It should be noticed that the mechanisms described so far for the function(s) played by CcmF (see [34, 36, 109]) do not envisage a clear role for its large C-terminal periplasmic domain (residues 510 to 611 in Ec-CcmF). It would be interesting to see if this domain, apparently devoid of recognizable sequence features, may mediate intermolecular recognition processes with one (or more) component(s) involved in the heme:apoCyt ligation process.

## 3. System II

System II is typically found in gram-positive bacteria and in in *ε*-proteobacteria; it is also present in most *β*- and some *δ*-proteobacteria, in *Aquificales* and cyanobacteria, as well as in algal and plant chloroplasts. System II is composed of three or four membrane-bound proteins: CcdA, ResA, CcsA (also known as ResC), and CcsB (also known as ResB) (Figure 3). CcdA and ResA are redox-active proteins involved in the reduction of the disulfide bond in the heme-binding motif of apoCyt, whereas CcsA and CcsB are responsible for the heme-apoCyt ligation process and are considered Cyt c synthethases (CCS). Both CcsA, which is evolutionary related to the CcmC and CcmF proteins of System I [55], and CcsB are integral membrane proteins. In some *ε*-proteobacteria, such as *Helicobacter hepaticus* and *Helicobacter pylori*, a single fusion protein composed of CcsA and CcsB polypeptides is present [41, 111]. Although, as discussed below, evidence has been put forward to support the hypothesis that the CcsBA complex acts a heme translocase, we still do not know if the heme is transported across the membrane by component(s) of System II itself or by a different, unidentified process.

### 3.1. System II: ApoCyt Thioreduction Pathway

After the Sec machinery secretes the newly synthesized apoCyt, it readily becomes a substrate for the oxidative system present on the outer surface of the cytoplasmic membrane of the gram-positive bacteria. In *B. subtilis,* the BdbCD system [112, 113] is functionally, but not structurally, equivalent to the well-characterized DsbAB system present in gram-negative bacteria. As discussed above for System I, the disulfide of the apoCyt heme-binding motif must be reduced in order to allow thioether bonds formation and heme attachment. ResA is the extracytoplasmic membrane-anchored TRX-like protein involved in the specific reduction of the apoCyt disulfide bond; after the disulfide bond exchange reaction has occurred, oxidized ResA is reduced by CcdA which receives its reducing equivalents from a cytoplasmic TRX [114]. Although ResA displays a classical TRX-like fold, the analysis of the 3D structure of oxidized and reduced ResA from *B. subtilis* (Bs-ResA) showed redox-dependent conformational modifications not observed in other TRX-like proteins. Interestingly, such modifications occur at the level of a cavity proposed to represent the binding site for oxidized apoCyt [24, 115, 116]. Another peculiar feature of Bs-ResA is represented by the unusually similar pK_*a*_ values of its the active site Cys residues (8.8 and 8.2, resp.), as observed also in the case of the active site Cys residues of the System I Pa-CcmH (see Section 2.3). However, at variance with Pa-CcmH, in Bs-ResA, the large separation between the two cysteine thiols observed in the structure of the reduced form of the protein can be invoked to account for this result.

CcdA is a large membrane protein containing six TM helices [117, 118], homologous to the TM domain of *E. coli* DsbD [85, 119]. Its role in the Cyt c biogenesis is supported by the observation that inactivation of CcdA blocks the production of c-type cytochromes in *B. subtilis* [120, 121]. Moreover, over and above the reduction of its apoCyt substrate, Bs-CcdA is able to reduce the disulfide bond of other secreted proteins, such as StoA [122]. It is interesting to note that ResA and CcdA are not essential for Cyt c synthesis in the absence of BdbD or BdbC or if a disulfide reductant is present in the growth medium [123, 124]. As discussed above (Section 2.4), a similar observation was made on the role of the thio-reductive pathway of System I: in this case, persistent production of c-type cytochromes was observed in Dsb-inactivated bacterial strains [38]. Following these observations, the question has been put forward as to why the Cyt c biogenesis process involves this apparently redundant thio-reduction route only to correct the effects of Bdb or Dsb activity. Referring to the case of *B. subtilis* [111], it has been proposed that the main reason is that BdbD must efficiently oxidize newly secreted proteins, since the activity of most of them depends on the presence of disulfide bonds. Alternatively, it can be hypothesized that the intramolecular disulfide bond of apoCyt protects the protein from proteolytic degradation or aggregation, or from cross-linking to other thiol-containing proteins.

### 3.2. System II: Heme Translocation and Attachment to ApoCyt

Recent experimental evidence is accumulating supporting the involvement of the heterodimeric membrane complex ResBC, or of the fusion protein CcsBA, in the translocation of heme from the n-side to the p-side of the bacterial membrane. In particular, it has been shown that CcsBA from *H. hepaticus* (Hh-CcsBA) is able to reconstitute Cyt c biogenesis in the periplasm of *E. coli* [36]. Moreover, it was also observed that Hh-CcsBA is able to bind reduced heme *via* two conserved histidines flanking the WWD domain and required both for the translocation of the heme and for the synthetase function of Hh-CcsBA [36]. A different study carried out on *B. subtilis* System II proteins provided support for heme-binding capability by the ResBC complex; according to the results obtained on recombinant ResBC, the ResB component of the heterodimeric CCS complex is able to covalently bind the heme in the cytoplasm (probably by a Cys residue) and to deliver it to an extracytoplamic domain of ResC, which is responsible for the covalent ligation to apoCyt [117]. It should be noticed, however, that the transfer of the CCS-bound heme to apoCyt still awaits direct experimental proof. Moreover, it has also been reported that in *B. subtilis*, the inactivation of CCS does not affect the presence of other heme-containing proteins in the periplasm, such as cytochrome b562 [111]; this observation, which is in contrast to the heme-transport hypothesis by CCSs, parallels similar concerns about heme translocation mechanism that are currently discussed in the context of System I (see Section 2.2 above). In the case of the Hh-CcsBA synthetase, site-directed mutagenesis experiments allowed to assign a heme-binding role for the two pairs of conserved His residues; according to the topological model of the protein obtained by alkaline phosphatase (PhoA) assays and GFP fusions, the conserved His77 and His858 residues are located on TM helices, while His761 and His897 are located on the periplasmic side [36]. These observations, together with the results of site-directed mutagenesis experiments, allowed the authors to suggest that His77 and His858 form a low affinity—membrane embedded, heme-binding site for ferrous heme which is subsequently translocated to an external heme-binding domain of Hh-CcsBA, where it is coordinated by His761 and His897. The topological model, therefore, predicts that this last His pair is part of a periplasmic-located WWD domain (homologous to the WWD domains found in the CcmC and CcmF proteins of System I described above).

## 4. System III

Strikingly different from Systems I and II, the Cyt c maturation apparatus found in fungi and in metazoan cells (System III) is composed of a single protein, known as HoloCytochrome c Synthetase (HCCS) or Cytochrome c Heme Lyase (CCHL), apparently responsible for all the subprocesses described above (heme transport and chaperoning, reduction and chaperoning of apoCyt, and catalysis of the thioether bonds formation between heme and apoCyt) (Figure 4). Surprisingly, although this protein has been identified in *S. cerevisiae* mitochondria several years ago [125, 126], only very recently the human HCCS has been expressed as a recombinant protein in *E. coli* and spectroscopically characterized *in vitro* [127]. It is worth noticing that human HCCS is attracting interest since, over and above its role in Cyt c biogenesis, it is involved in diseases such as microphthalmia with linear skin defects syndrome (MLS), an X-linked genetic disorder [128–130] and in other processes, such as Cytc-independent apoptosis in injured motor neurons [131]. Different from animal cells, where a single HCCS is sufficient for the maturation of both soluble and membrane-anchored Cytc (Cyt c and Cy c1, resp.), in *S. cerevisiae* two homologs, HCCS and HCC1S located in the inner mitochondrial membrane and facing to the IMS space [132, 133] are responsible for heme attachment to apoCyt c and apoCyt c1, respectively [125, 134]. It should also be noticed that in fungi, an additional FAD-containing protein (Cyc2p) is required for Cyt c synthesis. Cyc2p is a mitochondrial membrane-anchored flavoprotein exposing its redox domain to the IMS, which is required for the maturation of Cyt c but not for that of Cyt c1 [135]. This protein does not contain the conserved Cys residues typically found in disulfide reductases and indeed it is not able to reduce oxidized apoCyt *in vitro*. However, it has been recently shown that Cyc2p is able to catalyze the NAD(P)H-dependent reduction of heme *in vitro* [136], a necessary step before the thioether bond formation can occur, as discussed above (see Section 2.6). This result, together with the observation that Cyc2p interacts with HCCS and with apoCyt c and c1 lends support to the proposal that Cyc2p is involved in the reduction of the heme iron *in vivo* [136, 137].

Although HCCS proteins are crucial for Cyt c maturation, we still do not know how these proteins recognize their substrates (heme and apoCyt) and how they promote or catalyze the formation of thioether bonds between the heme vinyl groups and the cysteine thiols of the apoCyt CXXCH motif.

Contrary to the broad specificity of System I, which is able to recognize and attach the heme to prokaryotic and eukaryotic c-type cytochromes [49, 50], and even to very short microperoxidase-like sequences [49, 50], mitochondrial HCCS is characterized by a higher specificity, as it does not recognize bacterial apoCyt sequences. These observations prompted the investigation of the recognition process between apoCyt and HCCS. Recently, by using a recombinant yeast HCCS and chimerical apoCyt sequences expressed in *E. coli*, it was possible to conclude that a crucial recognition determinant is represented by the N-terminal region of apoCyt containing the heme-binding motif [35]; notice that, in the context of System I, the same N-terminal region of the Cyt c sequence has been recently identified to be important for the recognition by apoCcmE (see Section 2.6). Over and above the role played by the intervening residues in the CXXCH heme-binding motif [135], it has been shown that a conserved Phe residue, occurring in the N-terminal region before the CXXCH motif, is important for HCCS recognition [35, 138]. These results support the hypothesis that this residue, known to be a key determinant of Cytc folding and stability [19, 20, 139], may also be crucial for Cytc maturation by HCCS.

Another relevant question concerns the ability of HCCS to recognize and bind the heme molecule, as the region of the protein responsible for heme recognition remains to be identified. Initially, it was hypothesized that the recognition of heme could be mediated by the CP motifs present in the HCCS protein [140]; these short sequences are indeed known to bind heme in a variety of heme-containing proteins [141, 142]. However, it has been recently shown that CP motifs of the recombinant *S. cerevisiae* HCCS are not necessary for Cyt c production in *E. coli* [143], excluding them as key determinants of heme recognition.

The long-awaited *in vitro* characterization of the recombinant human HCCS allowed for the first time to propose a molecular mechanism underlying Cytc maturation in eukaryotes which can be experimentally tested [127]. According to the proposed model, the human HCCS activity can be described as a four step mechanism, involving (i) heme-binding, (ii) apoCyt recognition, (iii) thioether bonds formation, and (iv) holoCyt c release. In particular, the heme is proposed to play the role of a scaffolding molecule, mediating the contacts between HCCS and apoCyt. Mutagenesis experiments carried out on the recombinant HCCS strongly suggest that heme-binding (Step 1) depends on the presence of a specific His residue (His154), acting as an axial ligand to ferrous heme. Residues present at the N-terminus of apoCyt mediate the recognition with HCCS (Step 2); as discussed above, it is known that this region forms structurally conserved *α*-helix in the fold of all c-type cytochromes. Unfortunately, no information is available up to now concerning the region of HCCS involved in the recognition and binding to the N-terminal region of apoCyt. Coordination of the heme iron by the His residue of the apoCyt heme-binding motif CXXCH provides the second axial ligand to the heme iron and is probably important for the correct positioning of the two apoCyt Cys residues and formation of the thioether bonds (Step 3). The final release of functional Cyt c (Step 4) clearly requires the displacement of the His154:Fe^2+^ coordination bond; such a displacement is probably mediated by formation of the coordination bond with the Cyt c conserved Met residue and/or by the simultaneous folding of Cyt c. Again, it is interesting to note that this last hypothesis is in accordance with *in vitro* folding studies on c-type cytochromes that highlighted the late formation of the Met-Fe coordination during Cyt c folding [144, 145].

## Figures and Tables

**Table 1:** Protein components of Systems I, II, and III along with their structural features (or PDB codes, when known) and functional roles. System I proteins are found in *α*- and *γ*-proteobacteria, some *β*- and *δ*-proteobacteria, and *Deinococci* and *Archaea*. Plant mitochondria. Mitochondria of some protozoa. System II proteins are found in plant chloroplasts. Gram-positive bacteria, cyanobacteria, *ε*-proteobacteria, most *β*-proteobacteria, and some *δ*-proteobacteria. HCCS of System III is found in the mitochondria of fungi, vertebrates, and invertebrates.

System I (SI)	SI structural features	System II (SII)	SII structural features	System III (SIII)	SIII structural features	Function(s)
CcmA	ABC transporter; membrane n-side; nucleotide-binding domain	ResB (CcsB)	5-6 TM helices; 1 large periplasmic domain; conserved His residues	HCCS	Membrane-associated protein; conserved His residues	Heme translocation and delivery

CcmB	ABC transporter; 6 TM helices	ResC (CcsA)	6–8 TM helices; WWD domain; conserved His residues			

CcmC	6 TM helices; periplasmic WWD domain					

CcmD	Small membrane protein; 1 TM helix					

CcmE	1 TM helix; OB-fold 1SR3, 1LM0, 1J6Q, 2KCT					

CcmG	1 TM helix; TRX-like fold 1Z5Y, 2B1K, 1KNG, 3KH7, 3KH9, 3K8N	ResA	TRX-like fold 2H1B, 1SU9, 1ST9, 2F9S			apoCyt thioreduction

CcmH	1 TM helix; 3-helix bundle fold 2HL7, 2KW0	CcdA	6 TM helices			

CcmF	10–15 TM helices; periplasmic WWD domain; conserved His residues.	ResB (CcsB) ResC (CcsA)		HCCS		apoCyt chaperoning and heme attachment

CcmI	Periplasmic TPR and *α*/*β* domains					

## References

[1] Galinato MG, Kleingardner JG, Bowman SE (2012). Heme-protein vibrational couplings in cytochrome c provide a dynamic link that connects the heme-iron and the protein surface. *Proceedings of the National Academy of Sciences*.

[2] Gray HB, Winkler JR (2010). Electron flow through metalloproteins. *Biochimica et Biophysica Acta*.

[3] Ascenzi P, Santucci R, Coletta M, Polticelli F (2010). Cytochromes: reactivity of the “dark side” of the heme. *Biophysical Chemistry*.

[4] Yamada S, Bouley Ford ND, Keller GE, Ford WC, Gray HB, Winkler JR (2013). Snapshots of a protein folding intermediate. *Proceedings of the National Academy of Sciences*.

[5] Weinkam P, Zimmermann J, Romesberg FE, Wolynes PG (2010). The folding energy landscape and free energy excitations of cytochrome c. *Accounts of Chemical Research*.

[6] Yamanaka M, Masanari M, Sambongi Y (2011). Conferment of folding ability to a naturally unfolded apocytochrome c through introduction of hydrophobic amino acid residues. *Biochemistry*.

[7] Moore GW, Pettigrew GW (1990). *Cytochrome C: Evolutionary, Structural, and Physicochemical Aspects*.

[8] Bertini I, Cavallaro G, Rosato A (2006). Cytochrome c: occurrence and functions. *Chemical Reviews*.

[9] Pearce DA, Sherman F (1995). Degradation of cytochrome oxidase subunits in mutants of yeast lacking cytochrome c and suppression of the degradation by mutation of yme1. *Journal of Biological Chemistry*.

[10] Silkstone G, Kapetanaki SM, Husu I, Vos MH, Wilson MT (2012). Nitric oxide binding to the cardiolipin complex of ferric cytochrome c. *Biochemistry*.

[11] Giorgio M, Migliaccio E, Orsini F (2005). Electron transfer between cytochrome c and p66Shc generates reactive oxygen species that trigger mitochondrial apoptosis. *Cell*.

[12] Kilbride SM, Prehn JH (2013). Central roles of apoptotic proteins in mitochondrial function. *Oncogene*.

[13] Nöll T, Nöll G (2011). Strategies for “wiring” redox-active proteins to electrodes and applications in biosensors, biofuel cells, and nanotechnology. *Chemical Society Reviews*.

[14] Layer G, Reichelt J, Jahn D, Heinz DW (2010). Structure and function of enzymes in heme biosynthesis. *Protein Science*.

[15] Severance S, Hamza I (2009). Trafficking of heme and porphyrins in metazoa. *Chemical Reviews*.

[16] Hamel P, Corvest V, Giegé P, Bonnard G (2009). Biochemical requirements for the maturation of mitochondrial c-type cytochromes. *Biochimica et Biophysica Acta*.

[17] Fisher WR, Taniuchi H, Anfinsen CB (1973). On the role of heme in the formation of the structure of cytochrome c. *Journal of Biological Chemistry*.

[18] Yamanaka M, Mita H, Yamamoto Y, Sambongi Y (2009). Heme is not required for aquifex aeolicus cytochrome c555 polypeptide folding. *Bioscience, Biotechnology and Biochemistry*.

[19] Travaglini-Allocatelli C, Gianni S, Brunori M (2004). A common folding mechanism in the cytochrome c family. *Trends in Biochemical Sciences*.

[20] Borgia A, Bonivento D, Travaglini-Allocatelli C, Di Matteo A, Brunori M (2006). Unveiling a hidden folding intermediate in c-type cytochromes by protein engineering. *Journal of Biological Chemistry*.

[21] Enggist E, Thöny-Meyer L, Güntert P, Pervushin K (2002). NMR structure of the heme chaperone CcmE reveals a novel functional motif. *Structure*.

[22] di Matteo A, Calosci N, Gianni S, Jemth P, Brunori M, Travaglini-Allocatelli C (2010). Structural and functional characterization of CcmG from pseudomonas aeruginosa, a key component of the bacterial cytochrome c maturation apparatus. *Proteins*.

[23] di Matteo A, Gianni S, Schininà ME (2007). A strategic protein in cytochrome c maturation: three-dimensional structure of CcmH and binding to apocytochrome c. *Journal of Biological Chemistry*.

[24] Crow A, Acheson RM, Le Brun NE, Oubrie A (2004). Structural basis of redox-coupled protein substrate selection by the cytochrome c biosynthesis protein ResA. *Journal of Biological Chemistry*.

[25] di Silvio E, di Matteo A, Travaglini-Allocatelli C (2012). The Redox pathway of Pseudomonas aeruginosa cytochrome c biogenesis. *Journal of Proteins and Proteomics*.

[26] Thöny-Meyer L, Künzler P (1997). Translocation to the periplasm and signal sequence cleavage of preapocytochrome c depend on sec and lep, but not on the ccm gene products. *European Journal of Biochemistry*.

[27] Facey SJ, Kuhn A (2010). Biogenesis of bacterial inner-membrane proteins. *Cellular and Molecular Life Sciences*.

[28] Diekert K, de Kroon AI, Ahting U (2001). Apocytochrome c requires the TOM complex for translocation across the mitochondrial outer membrane. *The EMBO Journal*.

[29] Wiedemann N, Kozjak V, Prinz T (2003). Biogenesis of yeast mitochondrial cytochrome c: a unique relationship to the TOM machinery. *Journal of Molecular Biology*.

[30] Hartl F-U, Pfanner N, Neupert W (1987). Translocation intermediates on the import pathway of proteins into mitochondria. *Biochemical Society Transactions*.

[31] Glick BS, Brandt A, Cunningham K, Muller S, Hallberg RL, Schatz G (1992). Cytochromes c1 and b2 are sorted to the intermembrane space of yeast mitochondria by a stop-transfer mechanism. *Cell*.

[32] Howe G, Merchant S (1994). Role of heme in the biosynthesis of cytochrome c6. *Journal of Biological Chemistry*.

[33] Nakamoto SS, Hamel P, Merchant S (2000). Assembly of chloroplast cytochromes b and c. *Biochimie*.

[34] Sanders C, Turkarslan S, Lee D-W, Daldal F (2010). Cytochrome c biogenesis: the Ccm system. *Trends in Microbiology*.

[35] Stevens JM, Mavridou DA, Hamer R, Kritsiligkou P, Goddard AD, Ferguson SJ (2011). Cytochrome c biogenesis system I. *The FEBS Journal*.

[36] Kranz RG, Richard-Fogal C, Taylor J-S, Frawley ER (2009). Cytochrome c biogenesis: mechanisms for covalent modifications and trafficking of heme and for heme-iron redox control. *Microbiology and Molecular Biology Reviews*.

[37] Allen JWA (2011). Cytochrome c biogenesis in mitochondria—systems III and V. *The FEBS Journal*.

[38] Mavridou DA, Ferguson SJ, Stevens JM (2013). Cytochrome c assembly. *IUBMB Life*.

[39] Bertini I, Cavallaro G, Rosato A (2007). Evolution of mitochondrial-type cytochrome c domains and of the protein machinery for their assembly. *Journal of Inorganic Biochemistry*.

[40] Goldman BS, Kranz RG (1998). Evolution and horizontal transfer of an entire biosynthetic pathway for cytochrome c biogenesis: helicobacter, deinococcus, archae and more. *Molecular Microbiology*.

[41] Richard-Fogal CL, Frawley ER, Bonner ER, Zhu H, San Francisco B, Kranz RG (2009). A conserved haem redox and trafficking pathway for cofactor attachment. *The EMBO Journal*.

[42] Thony-Meyer L, Fischer F, Kunzler P, Ritz D, Hennecke H (1995). Escherichia coli genes required for cytochrome c maturation. *Journal of Bacteriology*.

[43] Beckett CS, Loughman JA, Karberg KA, Donato GM, Goldman WE, Kranz RG (2000). Four genes are required for the system II cytochrome c biogenesis pathway in Bordetella pertussis, a unique bacterial model. *Molecular Microbiology*.

[44] Le Brun NE, Bengtsson J, Hederstedt L (2000). Genes required for cytochrome c synthesis in Bacillus subtilis. *Molecular Microbiology*.

[45] Giegé P, Grienenberger JM, Bonnard G (2008). Cytochrome c biogenesis in mitochondria. *Mitochondrion*.

[46] Feissner RE, Richard-Fogal CL, Frawley ER, Loughman JA, Earley KW, Kranz RG (2006). Recombinant cytochromes c biogenesis systems I and II and analysis of haem delivery pathways in Escherichia coli. *Molecular Microbiology*.

[47] Cianciotto NP, Cornelis P, Baysse C (2005). Impact of the bacterial type I cytochrome c maturation system on different biological processes. *Molecular Microbiology*.

[48] Arslan E, Schulz H, Zufferey R, Künzler P, Thöny-Meyer L (1998). Overproduction of the Bradyrhizobium japonicum c-type cytochrome subunits of the cbb3 oxidase in Escherichia coli. *Biochemical and Biophysical Research Communications*.

[49] Sanders C, Lill H (2000). Expression of prokaryotic and eukaryotic cytochromes c in Escherichia coli. *Biochimica et Biophysica Acta*.

[50] Braun M, Thöny-Meyer L (2004). Biosynthesis of artificial microperoxidases by exploiting the secretion and cytochrome c maturation apparatuses of Escherichia coli. *Proceedings of the National Academy of Sciences of the United States of America*.

[51] Baert B, Baysse C, Matthijs S, Cornelis P (2008). Multiple phenotypic alterations caused by a c-type cytochrome maturation ccmC gene mutation in Pseudomonas aeruginosa. *Microbiology*.

[52] Turkarslan S, Sanders C, Daldal F (2006). Extracytoplasmic prosthetic group ligation to apoproteins: maturation of c-type cytochromes. *Molecular Microbiology*.

[53] Jones PM, George AM (2004). The ABC transporter structure and mechanism: perspectives on recent research. *Cellular and Molecular Life Sciences*.

[54] Christensen O, Harvat EM, Thöny-Meyer L, Ferguson SJ, Stevens JM (2007). Loss of ATP hydrolysis activity by CcmAB results in loss of c-type cytochrome synthesis and incomplete processing of CcmE. *The FEBS Journal*.

[55] Lee J-H, Harvat EM, Stevens JM, Ferguson SJ, Saier MH (2007). Evolutionary origins of members of a superfamily of integral membrane cytochrome c biogenesis proteins. *Biochimica et Biophysica Acta*.

[56] Beckman DL, Trawick DR, Kranz RG (1992). Bacterial cytochromes c biogenesis. *Genes and Development*.

[57] Schulz H, Fabianek RA, Pellicioli EC, Hennecke H, Thöny-Meyer L (1999). Heme transfer to the heme chaperone CcmE during cytochrome c maturation requires the CcmC protein, which may function independently of the ABC-transporter CcmAB. *Proceedings of the National Academy of Sciences of the United States of America*.

[58] Ren Q, Thöny-Meyer L (2001). Physical Interaction of CcmC with Heme and the Heme Chaperone CcmE during Cytochrome c Maturation. *Journal of Biological Chemistry*.

[59] Richard-Fogal C, Kranz RG (2010). The CcmC:heme:CcmE complex in heme trafficking and cytochrome c biosynthesis. *Journal of Molecular Biology*.

[60] Viswanathan VK, Kurtz S, Pedersen LL (2002). The cytochrome C maturation locus of Legionella pneumophila promotes iron assimilation and intracellular infection and contains a strain-specific insertion sequence element. *Infection and Immunity*.

[61] Ahuja U, Thöny-Meyer L (2005). CcmD is involved in complex formation between CcmC and the heme chaperone CcmE during cytochrome c maturation. *Journal of Biological Chemistry*.

[62] Richard-Fogal CL, Frawley ER, Kranz RG (2008). Topology and function of CcmD in cytochrome C maturation. *Journal of Bacteriology*.

[63] Schulz H, Hennecke H, Thöny-Meyer L (1998). Prototype of a heme chaperone essential for cytochrome c maturation. *Science*.

[64] Arnesano F, Banci L, Barker PD (2002). Solution structure and characterization of the heme chaperone CcmE. *Biochemistry*.

[65] Murzin AG (1993). OB(oligonucleotide/oligosaccharide binding)-fold: common structural and functional solution for non-homologous sequences. *The EMBO Journal*.

[66] Thöny-Meyer L (2003). A heme chaperone for cytochrome c biosynthesis. *Biochemistry*.

[67] Harvat EM, Redfield C, Stevens JM, Ferguson SJ (2009). Probing the heme-binding site of the cytochrome c maturation protein CcmE. *Biochemistry*.

[68] Lee D, Pervushin K, Bischof D, Braun M, Thöny-Meyer L (2005). Unusual heme-histidine bond in the active site of a chaperone. *Journal of the American Chemical Society*.

[69] Goddard AD, Stevens JM, Rao F (2010). c-Type cytochrome biogenesis can occur via a natural Ccm system lacking CcmH, CcmG, and the heme-binding histidine of CcmE. *Journal of Biological Chemistry*.

[70] Aramini JM, Hamilton K, Rossi P (2012). Solution NMR structure, backbone dynamics, and heme-binding properties of a novel cytochrome c maturation protein CcmE from Desulfovibrio vulgaris. *Biochemistry*.

[71] Uchida T, Stevens JM, Daltrop O (2004). The interaction of covalently bound heme with the cytochrome c maturation protein CcmE. *Journal of Biological Chemistry*.

[72] García-Rubio I, Braun M, Gromov I, Thöny-Meyer L, Schweiger A (2007). Axial coordination of heme in ferric CcmE chaperone characterized by EPR spectroscopy. *Biophysical Journal*.

[73] Thöny-Meyer L (2000). Haem-polypeptide interactions during cytochrome c maturation. *Biochimica et Biophysica Acta*.

[74] Keightley JA, Sanders D, Todaro TR, Pastuszyn A, Fee JA (1998). Cloning expression in Escherichia coli of the cytochrome c552 gene from Thermus thermophilus HB8. Evidence for genetic linkage to an ATP- binding cassette protein and initial characterization of the cycA gene products. *Journal of Biological Chemistry*.

[75] Monika EM, Goldman BS, Beckman DL, Kranz RG (1997). A thioreduction pathway tethered to the membrane for periplasmic cytochromes c biogenesis; In vitro and in vivo studies. *Journal of Molecular Biology*.

[76] Throne-Holst M, Thöny-Meyer L, Hederstedt L (1997). Escherichia coli ccm in-frame deletion mutants can produce periplasmic cytochrome b but not cytochrome c. *FEBS Letters*.

[77] Ahuja U, Thöny-Meyer L (2003). Dynamic features of a heme delivery system for cytochrome c maturation. *Journal of Biological Chemistry*.

[78] Allen JWA, Barker PD, Daltrop O (2005). Why isn’t “standard” heme good enough for c-type and di-type cytochromes?. *Dalton Transactions*.

[79] Inaba K, Ito K (2008). Structure and mechanisms of the DsbB-DsbA disulfide bond generation machine. *Biochimica et Biophysica Acta*.

[80] Kadokura H, Katzen F, Beckwith J (2003). Protein disulfide bond formation in prokaryotes. *Annual Review of Biochemistry*.

[81] Shouldice SR, Heras B, Walden PM, Totsika M, Schembri MA, Martin JL (2011). Structure and function of DsbA, a key bacterial oxidative folding catalyst. *Antioxidants and Redox Signaling*.

[82] Metheringham R, Griffiths L, Crooke H, Forsythe S, Cole J (1995). An essential role for DsbA in cytochrome c synthesis and formate-dependent nitrite reduction by Escherichia coli K-12. *Archives of Microbiology*.

[83] Sambongi Y, Ferguson SJ (1996). Mutants of Escherichia coli lacking disulphide oxidoreductases DsbA and DsbB cannot synthesise an exogenous monohaem c-type cytochrome except in the presence of disulphide compounds. *FEBS Letters*.

[84] Mavridou DA, Ferguson SJ, Stevens JM (2012). The interplay between the disulfide bond formation pathway and cytochrome c maturation in Escherichia coli. *FEBS Letters*.

[85] Stirnimann CU, Rozhkova A, Grauschopf U, Grütter MG, Glockshuber R, Capitani G (2005). Structural basis and kinetics of DsbD-dependent cytochrome c maturation. *Structure*.

[86] Ouyang N, Gao Y-G, Hu H-Y, Xia Z-X (2006). Crystal structures of E. coli CcmG and its mutants reveal key roles of the N-terminal *β*-sheet and the fingerprint region. *Proteins*.

[87] Edeling MA, Guddat LW, Fabianek RA (2001). Crystallization and preliminary diffraction studies of native and selenomethionine CcmG (CycY, DsbE). *Acta Crystallographica D*.

[88] Fabianek RA, Huber-Wunderlich M, Glockshuber R, Künzler P, Hennecke H, Thöny-Meyer L (1997). Characterization of the Bradyrhizobium japonicum CycY protein, a membrane-anchored periplasmic thioredoxin that may play a role as a reductant in the biogenesis of c-type cytochromes. *Journal of Biological Chemistry*.

[89] Kallis GB, Holmgren A (1980). Differential reactivity of the functional sulfhydryl groups of cysteine-32 and cysteine-32 present in the reduced form of thioredoxin from Escherichia coli. *Journal of Biological Chemistry*.

[90] Chivers PT, Raines RT (1997). General acid/base catalysis in the active site of Escherichia coli thioredoxin. *Biochemistry*.

[91] Zheng XM, Hong J, Li HY, Lin DH, Hu HY (2012). Biochemical properties and catalytic domain structure of the CcmH protein from Escherichia coli. *Biochim Biophys Acta*.

[92] Ahuja U, Rozhkova A, Glockshuber R, Thöny-Meyer L, Einsle O (2008). Helix swapping leads to dimerization of the N-terminal domain of the c-type cytochrome maturation protein CcmH from Escherichia coli. *FEBS Letters*.

[93] Fabianek RA, Hofer T, Thöny-Meyer L (1999). Characterization of the Escherichia coli CcmH protein reveals new insights into the redox pathway required for cytochrome c maturation. *Archives of Microbiology*.

[94] Reid E, Cole J, Eaves DJ (2001). The Escherichia coli CcmG protein fulfils a specific role in cytochrome c assembly. *Biochemical Journal*.

[95] Ren Q, Ahuja U, Thöny-Meyer L (2002). A bacterial cytochrome c heme lyase: CcmF forms a complex with the heme chaperone CcmE and CcmH but not with apocytochrome c. *Journal of Biological Chemistry*.

[96] Meyer EH, Giegé P, Gelhaye E (2005). AtCCMH, an essential component of the c-type cytochrome maturation pathway in Arabidopsis mitochondria, interacts with apocytochrome c. *Proceedings of the National Academy of Sciences of the United States of America*.

[97] Rios-Velazquez C, Coller R, Donohue TJ (2003). Features of Rhodobacter sphaeroides CcmFH. *Journal of Bacteriology*.

[98] Sanders C, Deshmukh M, Astor D, Kranz RG, Daldal F (2005). Overproduction of CcmG and CcmFHRc fully suppresses the c-type cytochrome biogenesis defect of Rhodobacter capsulatus CcmI-null mutants. *Journal of Bacteriology*.

[99] Verissimo AF, Yang H, Wu X, Sanders C, Daldal F (2011). CcmI subunit of CcmFHI heme ligation complex functions as an apocytochrome c chaperone during c-type cytochrome maturation. *Journal of Biological Chemistry*.

[100] Bryson K, McGuffin LJ, Marsden RL, Ward JJ, Sodhi JS, Jones DT (2005). Protein structure prediction servers at University College London. *Nucleic Acids Research*.

[101] Jones DT (1999). Protein secondary structure prediction based on position-specific scoring matrices. *Journal of Molecular Biology*.

[102] Sanders C, Boulay C, Daldal F (2007). Membrane-spanning and periplasmic segments of CcmI have distinct functions during cytochrome c biogenesis in Rhodobacter capsulatus. *Journal of Bacteriology*.

[103] Han D, Kim K, Oh J, Park J, Kim Y (2008). TPR domain of NrfG mediates complex formation between heme lyase and formate-dependent nitrite reductase in Escherichia coli O157:H7. *Proteins*.

[104] Eaves DJ, Grove J, Staudenmann W (1998). Involvement of products of the nrfEFG genes in the covalent attachment of haem c to a novel cysteine-lysine motif in the cytochrome c552 nitrite reductase from Escherichia coli. *Molecular Microbiology*.

[105] Nilsson J, Persson B, Von Heijne G (2000). Consensus predictions of membrane protein topology. *FEBS Letters*.

[106] di Silvio E, di Matteo A, Malatesta F, Travaglini-Allocatelli C (2013). Recognition and binding of apocytochrome c to P. aeruginosa CcmI, a component of cytochrome c maturation machinery. *Biochim Biophys Acta*.

[107] Zeytuni N, Zarivach R (2012). Structural and functional discussion of the tetra-trico-peptide repeat, a protein interaction module. *Structure*.

[108] Sanders C, Turkarslan S, Lee D-W, Onder O, Kranz RG, Daldal F (2008). The cytochrome c maturation components CcmF, CcmH, and CcmI form a membrane-integral multisubunit heme ligation complex. *Journal of Biological Chemistry*.

[109] Verissimo AF, Mohtar MA, Daldal F (2013). The heme chaperone ApoCcmE forms a ternary complex with CcmI and apocytochrome c. *The Journal of Biological Chemistry*.

[110] Barker PD, Ferrer JC, Mylrajan M (1993). Transmutation of a heme protein. *Proceedings of the National Academy of Sciences of the United States of America*.

[111] Simon J, Hederstedt L (2011). Composition and function of cytochrome c biogenesis system II. *The FEBS Journal*.

[112] Kouwen TRHM, van Dijl JM (2009). Interchangeable modules in bacterial thiol-disulfide exchange pathways. *Trends in Microbiology*.

[113] Crow A, Lewin A, Hecht O (2009). Crystal structure and biophysical properties of Bacillus subtilis BdbD. An oxidizing thiol:disulfide oxidoreductase containing a novel metal site. *Journal of Biological Chemistry*.

[114] Möller MC, Hederstedt L (2008). Extracytoplasmic processes impaired by inactivation of trxA (thioredoxin gene) in Bacillus subtilis. *Journal of Bacteriology*.

[115] Lewin A, Crow A, Oubrie A, Le Brun NE (2006). Molecular basis for specificity of the extracytoplasmic thioredoxin ResA. *Journal of Biological Chemistry*.

[116] Colbert CL, Wu Q, Erbel PJA, Gardner KH, Deisenhofer J (2006). Mechanism of substrate specificity in Bacillus subtilis ResA, a thioredoxin-like protein involved in cytochrome c maturation. *Proceedings of the National Academy of Sciences of the United States of America*.

[117] Ahuja U, Kjelgaard P, Schulz BL, Thony-Meyer L, Hederstedt L (2009). Haem-delivery proteins in cytochrome c maturation system II. *Molecular Microbiology*.

[118] Page MLD, Hamel PP, Gabilly ST (2004). A homolog of prokaryotic thiol disulfide transporter CcdA is required for the assembly of the cytochrome b6f complex in Arabidopsis chloroplasts. *Journal of Biological Chemistry*.

[119] Rozhkova A, Stirnimann CU, Frei P (2004). Structural basis and kinetics of inter- and intramolecular disulfide exchange in the redox catalyst DsbD. *The EMBO Journal*.

[120] Schiött T, Throne-Holst M, Hederstedt L (1997). Bacillus subtilis CcdA-defective mutants are blocked in a late step of cytochrome C biogenesis. *Journal of Bacteriology*.

[121] Feissner RE, Beckett CS, Loughman JA, Kranz RG (2005). Mutations in cytochrome assembly and periplasmic redox pathways in Bordetella pertussis. *Journal of Bacteriology*.

[122] Erlendsson LS, Möller M, Hederstedt L (2004). Bacillus subtilis StoA is a thiol-disulfide oxidoreductase important for spore cortex synthesis. *Journal of Bacteriology*.

[123] Erlendsson LS, Acheson RM, Hederstedt L, Le Brun NE (2003). Bacillus subtilis ResA is a thiol-disulfide oxidoreductase involved in cytochrome c synthesis. *Journal of Biological Chemistry*.

[124] Erlendsson LS, Hederstedt L (2002). Mutations in the thiol-disulfide oxidoreductases BdbC and BdbD can suppress cytochrome c deficiency of CcdA-defective Bacillus subtilis cells. *Journal of Bacteriology*.

[125] Dumont ME, Ernst JF, Hampsey DM, Sherman F (1987). Identification and sequence of the gene encoding cytochrome c heme lyase in the yeast Saccharomyces cerevisiae. *The EMBO Journal*.

[126] Dumont ME, Ernst JF, Sherman F (1988). Coupling of heme attachment to import of cytochrome c into yeast mitochondria. Studies with heme lyase-deficient mitochondria and altered apocytochromes c. *Journal of Biological Chemistry*.

[127] San Francisco B, Bretsnyder EC, Kranz RG (2012). Human mitochondrial holocytochrome c synthase's heme binding, maturation determinants, and complex formation with cytochrome c. *Proceedings of the National Academy of Sciences of the United States of America*.

[128] Schaefer L, Ballabio A, Zoghbi HY (1996). Cloning and characterization of a putative human holocytochrome c-type synthetase gene (HCCS) isolated from the critical region for microphthalmia with linear skin defects (MLS). *Genomics*.

[129] Prakash SK, Cormier TA, McCall AE (2002). Loss of holocytochrome c-type synthetase causes the male lethality of X-linked dominant microphthalmia with linear skin defects (MLS) syndrome. *Human Molecular Genetics*.

[130] Wimplinger I, Morleo M, Rosenberger G (2006). Mutations of the mitochondrial holocytochrome c-type synthase in X-linked dominant microphthalmia with linear skin defects syndrome. *The American Journal of Human Genetics*.

[131] Kiryu-Seo S, Gamo K, Tachibana T, Tanaka K, Kiyama H (2006). Unique anti-apoptotic activity of EAAC1 in injured motor neurons. *The EMBO Journal*.

[132] Lill R, Stuart RA, Drygas ME, Nargang FE, Neupert W (1992). Import of cytochrome c heme lyase into mitochondria: a novel pathway into the intermembrane space. *The EMBO Journal*.

[133] Steiner H, Zollner A, Haid A, Neupert W, Lill R (1995). Biogenesis of mitochondrial heme lyases in yeast. Import and folding in the intermembrane space. *Journal of Biological Chemistry*.

[134] Zollner A, Rodel G, Haid A (1992). Molecular cloning and characterization of the *Saccharomyces cerevisiae CYT2* gene encoding cytochrome-*c1*-heme lyase. *European Journal of Biochemistry*.

[135] Corvest V, Murrey DA, Bernard DG, Knaff DB, Guiard B, Hamel PP (2010). *c*-Type cytochrome assembly in *Saccharomyces cerevisiae*: a key residue for apocytochrome *c1*/lyase interaction. *Genetics*.

[136] Corvest V, Murrey DA, Hirasawa M, Knaff DB, Guiard B, Hamel PP (2012). The flavoprotein Cyc2p, a mitochondrial cytochrome c assembly factor, is a NAD(P)H-dependent haem reductase. *Molecular Microbiology*.

[137] Bernard DG, Quevillon-Cheruel S, Merchant S, Guiard B, Hamel PP (2005). Cyc2p, a membrane-bound flavoprotein involved in the maturation of mitochondrial c-type cytochromes. *Journal of Biological Chemistry*.

[138] Kleingardner JG, Bren KL (2011). Comparing substrate specificity between cytochrome c maturation and cytochrome c heme lyase systems for cytochrome c biogenesis. *Metallomics*.

[139] Travaglini-Allocatelli C, Gianni S, Morea V, Tramontano A, Soulimane T, Brunori M (2003). Exploring the cytochrome c folding mechanism: cytochrome c552 from Thermus thermophilus folds through an on-pathway intermediate. *Journal of Biological Chemistry*.

[140] Zhang L, Guarente L (1995). Heme binds to a short sequence that serves a regulatory function in diverse proteins. *The EMBO Journal*.

[141] Hira S, Tomita T, Matsui T, Igarashi K, Ikeda-Saito M (2007). Bach1, a heme-dependent transcription factor, reveals presence of multiple heme binding sites with distinct coordination structure. *IUBMB Life*.

[142] Kühl TK, Wißbrock A, Goradia N (2013). Analysis of Fe(III) heme binding to cysteine-containing heme-regulatory motifs in proteins. *ACS Chemical Biology*.

[143] Moore RL, Stevens JM, Ferguson SJ (2011). Mitochondrial cytochrome c synthase: CP motifs are not necessary for heme attachment to apocytochrome c. *FEBS Letters*.

[144] Gianni S, Brunori M, Travaglini-Allocatelli C (2001). Refolding kinetics of cytochrome c551 reveals a mechanistic difference between urea and guanidine. *Protein Science*.

[145] Gianni S, Travaglini-Allocatelli C, Cutruzzolà F, Brunori M, Shastry MCR, Roder H (2003). Parallel pathways in cytochrome c551 folding. *Journal of Molecular Biology*.

